# Chitosan Nanoparticles with Encapsulated Natural and UF-Purified Annatto and Saffron for the Preparation of UV Protective Cosmetic Emulsions

**DOI:** 10.3390/molecules23092107

**Published:** 2018-08-22

**Authors:** Sonia Ntohogian, Viktoria Gavriliadou, Evi Christodoulou, Stavroula Nanaki, Smaro Lykidou, Panagiotis Naidis, Lily Mischopoulou, Panagiotis Barmpalexis, Nikolaos Nikolaidis, Dimitrios N. Bikiaris

**Affiliations:** 1 Laboratory of Chemistry and Technology of Polymers and Dyes, Department of Chemistry, Aristotle University of Thessaloniki, 54124 Thessaloniki, Greece; sonita_nt@hotmail.gr (S.N.); victoria.vgche@gmail.com (V.G.); evicius@gmail.com (E.C.); sgnanaki@chem.auth.gr (S.N.); slykidou@chem.auth.gr (S.L.); pan-naidis@hotmail.com (P.N.); magoo-mm@hotmail.com (L.M.); 2 Department of Pharmaceutical Technology, School of Pharmacy, Aristotle University of Thessaloniki, 54124 Thessaloniki, Greece; pbarmp@pharm.auth.gr

**Keywords:** chitosan, nanoparticles, sunscreen, annatto, saffron, sun protection factor

## Abstract

The aim of the present work is to evaluate the preparation of sunscreen emulsions based on chitosan (CS) nanoparticles with annatto, ultrafiltrated (UF) annatto, saffron, and ultrafiltrated saffron. Ionic gelation was used for the preparation of chitosan nanoparticles, while their morphological characteristics and physicochemical properties were evaluated via Fourier transform infrared (FTIR) spectroscopy, X-ray diffraction (XRD) analysis, scanning electron microscopy (SEM), and dynamic light scattering (DLS). Results showed that the prepared nanoparticles ranged from ~150 to ~500 nm and had a spherical or irregular shape. In the case of annatto and UF annatto, due to the formation of H-bonds, the sunscreen agents were amorphously dispersed within CS nanoparticles, while in the case of saffron and UF saffron, crystalline dispersion was observed. All encapsulated materials had good thermal stability as well as color stability. In a further step, sunscreen emulsions were prepared based on the formed CS-sunscreen nanoparticles and evaluated for their stability in terms of pH and viscosity, along with their ultraviolet (UV) radiation protection ability in terms of sun protection factor (SPF). All prepared emulsions showed low cytotoxicity and good storage stability for up to 90 days, while minimum sunscreen protection was observed with SPF values varying from 2.15 to 4.85.

## 1. Introduction

Today, it is well documented that many of the adverse effects resulting from the exposure of skin to solar radiation are mainly caused by ultraviolet (UV) radiation from solar rays. Specifically, although UV-C radiation (100–290 nm) is totally filtered out by the earth’s ozone layer, UV-A (320–400 nm) and UV-B (290–320 nm) radiations are responsible for several skin pathologies such as sunburns, cutaneous degeneration, photosensitivity, phototoxicity, photoaging, immunosuppression, and skin cancer [1,2]. In order to prevent these adverse reactions, sunscreens that contain filter substances with strong protective efficacy against UV radiation are widely used.

Sun protection creams, lotions, oils, emulsions, and textiles are common choices of cosmetic/wear products used for sun protection, while materials of either physical or chemical nature are commonly selected as sun protecting (sunscreen) agents [3]. These agents act by absorbing/scattering or attenuating the solar UV radiation, respectively [4]. The efficacy of a sunscreen agent or the resultant sun protection product is usually expressed in terms of sun protection factor (SPF), where generally, a higher SPF value corresponds to more effective protection [5,6,7]. Several problems related to sunscreen products currently available on the market include high opacity with unacceptable appearance on the skin, erythema, edema, and irritation, among others [3]. In order to overcome these drawbacks, formulators have turned their attention to nanotechnology by designing formulas in which appropriate carriers work as vehicles for carrying nanosized sunscreen agents (such as zinc and titanium dioxide), by which more effective and cosmetically acceptable sunscreen products are prepared [3,8,9]. One such highly effective biocompatible material, used as a nanocarrier in the production of sun protection products, is chitosan (CS) [10,11].

CS is a polysaccharide of *N*-acetyl-d-glucosamine and d-glucosamine consisting of polymeric (1→4)-linked 2-amino-2-deoxy-β-d-glucopyranose units produced by the deacetylation of chitin [12,13,14]. CS is a biocompatible, nontoxic, and biodegradable material and shows good mucoadhesive and membrane-permeability-enhancing properties [15,16,17,18,19]. Due to several advantages, CS-based nanoparticles have been extensively evaluated as absorption enhancers (for drugs, peptides, and proteins) and as carriers in various drug delivery systems for oral, ocular, gene delivery, etc. [14,19,20,21,22,23,24,25,26,27]. There are several methods available for CS nanoparticle preparation including ionotropic gelation, microemulsification, emulsification solvent diffusion, and polyelectrolyte complexing [28]. Out of these, ionotropic gelation offers many advantages, such as simple and mild preparation without the use of organic solvents or high shear forces [13,28,29,30,31,32].

Nowadays, it is widely recognized that among the reasons for the development of severe skin damages due to sun exposure is also the production of free radicals [33]. Recently, it was demonstrated that 50% of free radicals are produced by solar radiation in the visible (VIS) and infrared (IR) spectral region [34]. Therefore, for ultimate skin protection, new concepts need to emerge that can effectively block the radiation of the earth’s complete solar spectrum (UV, VIS, and IR spectral regions). Such approaches may utilize the application of pigments and antioxidants [34], where, in the former case, the sunscreen agent acts as micromirror in the human skin, reflecting the sunlight not only in the UV but also in the VIS/IR spectral regions [35,36]. Such naturally occurring sunscreen agents that can combine both pigment and antioxidant properties may come from plant extracts such as green tea polyphenols, silymarin from milk thistle, proanthocyanidins from grape seeds, etc. [37,38]. Annatto, which comes from the family of Bixaceae (Achiote family), grows to a height of 2–5 m and is well distributed in Karnataka, Andhra Pradesh, Assam, Tamil Nadu, and Orissa (India). The dye coming from annatto is basically a red-orange pigment known as bixin and norbixin (carotenoids) and is extracted from the seed coat, which is inside a prickly heart-shaped pod, and is used in food, cosmetic, and soap industries as pigment [39]. Additionally, annatto’s carotenoids are considered as powerful antioxidant compounds [40]. Saffron is the dried red/yellow stigmas of a flower scientifically identified as *Crocus sativus* L, which is cultivated in Iran and some other countries such as India, Spain, and Greece. Pharmacological studies have been revealed that saffron extracts or its constituents have antitumor, hypolipidemic, radical scavenging, antinociceptive, anti-inflammatory, anticonvulsant, antidepressant, and antioxidant properties [41,42,43,44,45]. Therefore, due to the combination of pigment and antioxidant properties, several attempts have been made in order to prepare sunscreen formulations based on annatto or saffron [38,39,46,47].

Hence, the aim of the present study is to prepare new CS-based nanoparticles loaded with annatto or saffron and to evaluate their applicability in the preparation of new sunscreen products. For this reason, CS nanoparticles loaded with natural and ultrafiltrated (UF) annatto or saffron, prepared by ionotropic gelation, will be evaluated, while the SPF and stability of a new sunscreen emulsion will be accessed.

## 2. Results and Discussion

### 2.1. CS-Based Nanoparticles

**Figure 1:**
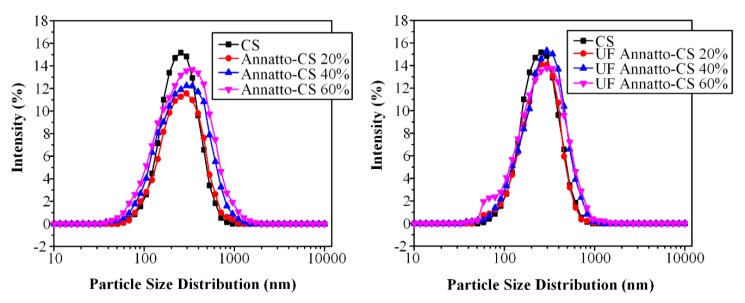
Particle size distribution measured by dynamic light scattering (DLS) of chitosan (CS)-loaded nanoparticles with annatto and ultrafiltrated (UF) annatto.

Generally, during ionotropic gelation, the formation of CS nanoparticles depends mainly on the ionic interaction of CS with tripolyphosphate (TPP), which eventually leads to the reduction of CS aqueous solubility. The effect of various ratios between CS and TPP on the size and particle distribution of CS nanoparticles was extensively studied in previous works [14]. Results indicated that good nanoparticles with a relatively low polydispersity index may result from CS:TPP ratios varying from 2:1 up to 5:1. Hence, in the present study, CS nanoparticles were prepared at a 2:1 CS-to-TPP ratio. In order to evaluate the particle size and morphology of the prepared nanoparticles, SEM was used. Figure 1 shows the obtained dynamic light scattering (DLS) data of the blank CS nanoparticles and the prepared CS-loaded annatto, UF annatto, saffron, and UF saffron nanoparticles. For the blank sample, nanoparticles with a mean particle size of 250 μm were produced (Table 1), which was verified from DLS measurements and SEM micrographs.

From the obtained SEM micrographs (data not shown), it is seen that both annatto (or UF annatto) and saffron (or UF saffron) nanoparticle size and morphology did not differ compared to the blank CS nanoparticles, indicating that the addition of the sunscreen protective agents did not affect the nanoparticle preparation method. In regards to annatto and UF-annatto–CS nanoparticles, the particle size ranged from ~170 to ~450 nm and had a mixed spherical and irregular shape, while the use of physical and UF annatto did not show any substantial differences regarding the size and shape of the resultant nanoparticles. Additionally, the varying annatto concentration (20–60% *w*/*w*) did not alter the morphological characteristics of the obtained particles. Furthermore, in the case of saffron and UF-saffron–CS-prepared nanoparticles, particle size varied from ~150 to ~500 nm, while the shape of the obtained nanoparticles was similar to that of annatto (or UF annatto)–CS nanoparticles. In order to have a better idea regarding the particle size distribution of the prepared nanoparticles, DLS measurements were conducted and the results are presented in Figure 1. 

As can be seen, in all nanoparticles, there is a broad size distribution varying from about 40 to 1000–1100 nm. When annatto was encapsulated in CS nanoparticles, the D(50) value is slightly increased compared to nanoparticles with neat CS (from 250 to 287–340 nm), while nanoparticle size increases with increasing annatto’s concentration (Table 1). This is probably because annatto has no reactive amino group that could ionically interact with TPP and thus annatto has a hindering effect to form ionically crosslinked gels. A similar trend was also observed when UF annatto was encapsulated in CS nanoparticles, where slightly lower D(50) values where observed compared to nanoparticles with annatto (probably due to UF annatto’s higher solubility). The zeta potential of chitosan nanoparticles is about 36 ± 3 mV, which is in agreement with the reported values for ionically crosslinked CS nanoparticles with TPP [48]. In encapsulated CS/annatto nanoparticles, the zeta potential remains positive, while their values were slightly increased compared to neat CS nanoparticles (Table 1). Similar results were also recorded for saffron and UF-saffron–CS-based nanoparticles (Table 1).

**Figure 2:**
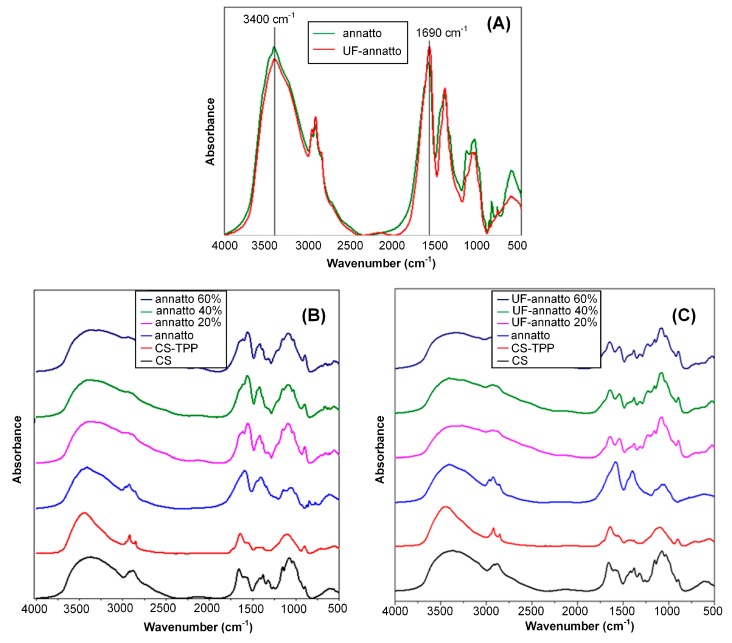
FTIR spectra of: (**A**) annatto and UF annatto; (**B**) neat CS, CS/tripolyphosphate (TPP) and annatto–CS nanoparticles; and (**C**) UF-annatto–CS nanoparticles.

In a further step, FTIR analysis was used in order to identify any possible interactions taking place between the sunscreen agents and CS, since any kind of physicochemical interaction will automatically lead to frequency shifts or splitting in the observed absorption peaks compared to the peaks of the neat compounds. Figure 2 shows the FTIR spectra of the prepared annatto and UF-annatto–CS nanoparticles. In regards to pure CS, several characteristic absorbance peaks were identified at: (1) 3400 cm^−1^, where the characteristic peak of the hydroxyl group m(OH) is recorded along with the overlapped peak of N–H stretch at 3278 cm^−1^; at (2) 1657 and 1594 cm^−1^, where the characteristic peaks of amide I and amide II bands are depicted, respectively; at (3) 1322 cm^−1^, where the characteristic peak of C–N stretch is recorded; and at (4) 1079 cm^−1^, where the characteristic peak of C–O stretch is recorded. In the case of CS–TPP nanoparticles, the FTIR peaks corresponding to the amino group’s absorption is shifted to 1640 and 1530 cm^−1^, indicating that these groups are probably interacting with TPP by creating ionic bonds. Additionally, a shift in the hydroxyl group band is recorded with a maximum at 3416 cm^−1^, indicating possible formation of H-bonds. In the case of annatto and UF annatto, the FTIR spectra display a broadband at 3400 cm^−1^ (ascribed to the stretching vibration of the –OH group), at 2920 and 2855 cm^−1^ (associated with the stretching vibrations of the hydrocarbon skeleton), and at 1690, 1603, and 1149 cm^−1^ (assigned to the stretching vibration of the C=O group, the conjugated C=C group, and the –CO of the carboxylic acid group).

All these absorption peaks can be ascribed to the carotenoid compounds of the annatto (bixin and norbixin). Additionally, no significant spectral differences were observed between the annatto and the UF annatto, indicating the UF process did not alter the chemical characteristics of the compound. In regards to annatto–CS nanoparticles, FTIR analysis showed that in the region of the hydroxyl group stretching (3600–3000 cm^−1^), a broader peak is observed compared to the neat compounds, indicating that probably some H-bonds are taking place between the hydroxyl or amino groups of CS with the carboxyl groups of annatto or UF annatto. Additionally, analysis showed no significant spectral differences between the nanoparticles containing different amounts of annatto or UF annatto (20%, 40%, and 60%), indicating that the same interactions (H-bonds) with CS are occurring independently of annatto (or UF annatto) concentration, while small differences in the intensity of the observed peaks may be attributed to the different encapsulation percentage of annatto (or UF annatto). 

**Figure 3:**
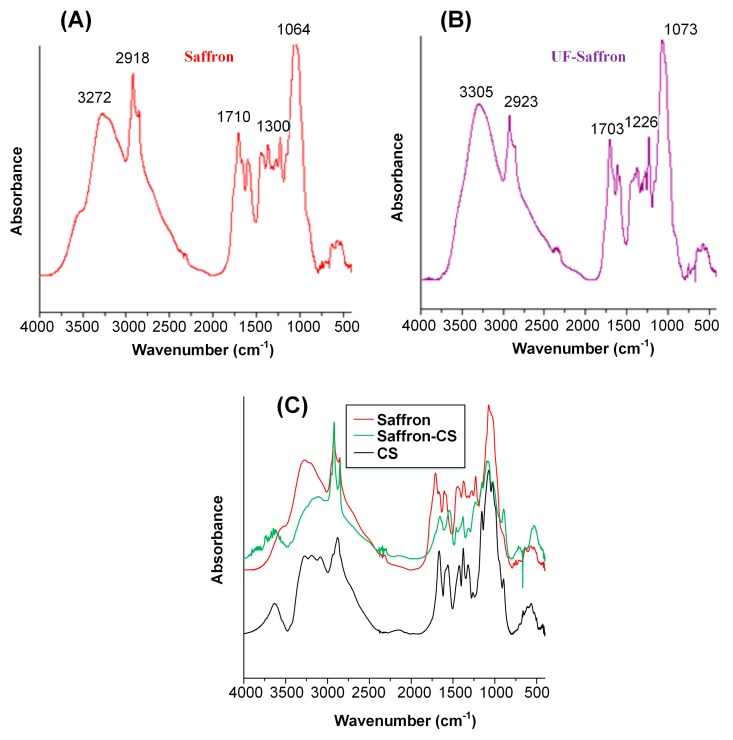
FTIR spectra of saffron (**A**), UF saffron (**B**), and saffron–CS nanoparticles (**C**).

Figure 3 shows the FTIR spectra of the prepared saffron–CS nanoparticles. Analysis of the saffron (or UF saffron) FTIR spectra did not show any significant differences between the two compounds, indicating that the UF process followed, although results in a more purified compound do not alter the chemical structure of saffron, while the spectra of both compounds showed several characteristic peaks [49]. 

Specifically, the band at ~3300 cm^−1^ is due to the stretching vibration of O–H, which indicates the presence of alcoholic groups. The two peaks in the region of 3000–2750 cm^−1^ correspond to C–H stretching vibration, which is indicative of the aldehyde group found in volatile components of saffron such as safranal. The C=O stretching vibration was found at ~1700 cm^−1^ in the spectra for saffron and UF saffron, while the presence of characteristic bands in the region of 1300–1220 cm^−1^ are due to stretching vibration of ester (O=C–O) groups, which are due to constituents of saffron such as dimethylcrocetin as well as alcohol groups found in the carbohydrate moiety of crocin esters and picrocrocin. The strong peak at 1100–1000 cm^−1^ is due to the stretching vibration mode of conjugated C–C bonds of the central carotenoid chain, which is characteristic for carotenoids such as crocetin esters. This peak might also be a characteristic of pyranose moiety, with multichain peaks related to the sequential arrangement of hydroxyl groups. In regards to the saffron–CS nanoparticles’ FTIR spectra, significant differences were observed at 3400–3000 cm^−1^ (region where characteristic peaks of both O–H and N–H groups are observed) and at 1594 cm^−1^, where the characteristic peak of amide II for CS shifts to lower wavenumbers, indicating the presence of significant interactions (probably H-bonds) between the two compounds. Similar differences were also observed in the case of UF-saffron–CS nanoparticles (data not shown).

**Figure 4:**
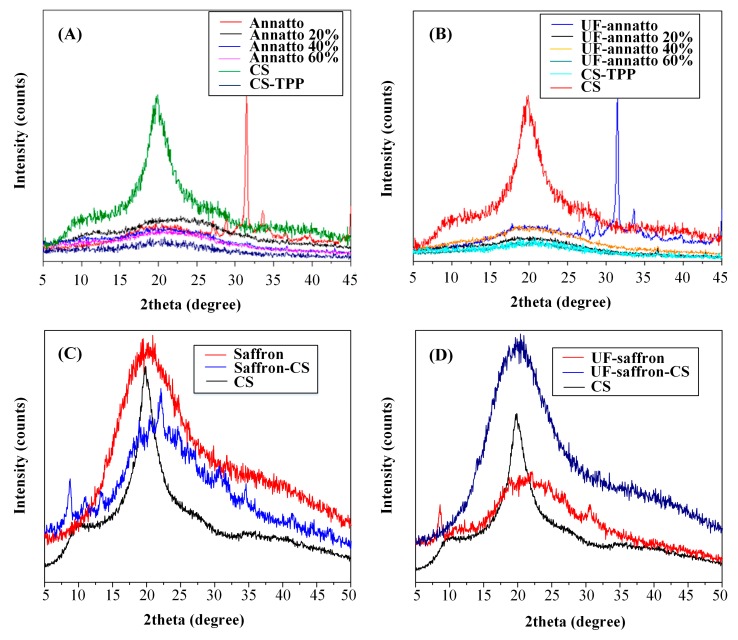
XRD diffractograms of: (**A**) CS, CS–TPP, annatto and annatto-CS nanoparticles; (**B**) UF-annatto and UF-annatto-CS nanoparticles; (**C**) saffron and saffron-CS nanoparticles; (**D**) UF-saffron and UF-saffron-CS nanoparticles.

In a further step, in order to identify the physical state of the tested naturally occurring sunscreen agents within the CS nanoparticle system, wide angle X-ray diffractometry was used. Figure 4 shows the XRD diffractograms of the neat components and the prepared nanoparticles. In regards to neat CS, XRD analysis showed two broad peaks at 2θ = 11° and mainly at 21°, verifying the semicrystalline nature of the polymer. When CS–TPP nanoparticles have been prepared, these are completely amorphous, since only a broad halo was recorded. Annatto showed high crystallinity, with XRD peaks recorded at 2θ of 27°, 29°, 34°, 36°, and 40°, while no differences were observed between the XRD patterns of annatto and UF annatto, indicating that no polymeric transition occurs during the UF process. On the other hand, both neat saffron and UF saffron showed similar XRD patterns, with an amorphous halo at ~20° indicating that the sunscreen agents are amorphous.

In regards to annatto–CS and UF-annatto–CS nanoparticles, XRD analysis showed that all prepared systems were amorphous, as no annatto- or UF-annatto-related XRD peaks were recorded. Additionally, increasing annatto (or UF annatto) concentration did not result in any significant change of the XRD patterns, expect in the reduction of the intensity of the broad peak of CS at 21° (due to lower CS concentration). The observed amorphization of annatto and UF annatto in the prepared CS nanoparticles may be attributed to the interactions observed by FTIR analysis (H-bonds). In the case of saffron and UF-saffron–CS nanoparticles, XRD analysis showed the presence of several small crystalline peaks at approximately 2θ of 8°, 11°, 18°, 22.5°, and 30° along with a characteristic amorphous halo. Since saffron is completely amorphous, these small peaks indicate that CS was transformed during nanoencapsulation into another crystalline form, which is in good agreement with the literature [50]. 

In regards to nanoparticle yields, UF-saffron–CS nanoparticles showed the highest yield, with a value of 83.75%, followed by annatto–CS nanoparticles, with a yield of 70.83%, while the lowest yield was obtained for UF-annatto–CS, with a value of 46.34% (Table 1). Additionally, in the case of annatto–CS nanoparticles, increasing amounts of annatto led to decreasing nanoparticle yield values, while the use of UF annatto led to opposite results, with increasing yields at higher sunscreen concentrations. In the case of sunscreen loadings, results from Table 1 show that increasing annatto and UF annatto concentrations led to increasing loading percentages. In saffron and UF saffron nanoparticles, the results showed a lower loading presentence, perhaps due to the higher solubility of saffron compared with annatto. Finally, increased entrapment efficiency values were observed in the case of most of the annatto and UF-annatto–CS nanoparticles, while low entrapment was observed in the case of saffron and UF-saffron–CS nanoparticles (Table 1). 

**Figure 5:**
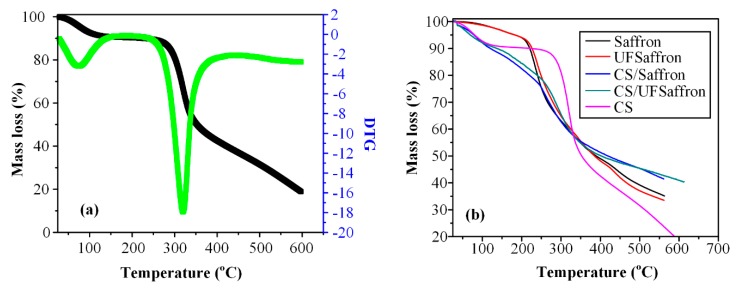
Thermogravimetric analysis (TGA) curves of neat CS (**a**) and the encapsulated materials on CS containing 40 wt% saffron or UF saffron (**b**).

The thermal stability of the prepared materials was evaluated via thermogravimetric analysis (TGA). As can be seen form Figure 5, chitosan has two main decomposition steps (Figure 5a). The first, corresponding to adsorbed moisture, takes place between ambient temperature and 120 °C [51]. The second takes place at much higher temperatures, with maximum mass loss at 320 °C, corresponding to the decomposition of polysaccharide macromolecular chains. Saffron and UF saffron have similar decomposition behaviour since both are natural products. There is a gradually small mass loss till 200 °C due to the absorbed water and, after that, the main decomposition takes place with a maximum decomposition at 250 °C (Figure 5b). In the case of encapsulated CS/saffron and CS/UF saffron, as expected, the thermal behaviour lies between the neat materials. At initial stages, absorbed water is volatised gradually till 200 °C, and after that point, the main decomposition takes place, which lies between that of saffron and CS (closer to saffron).

**Figure 6:**
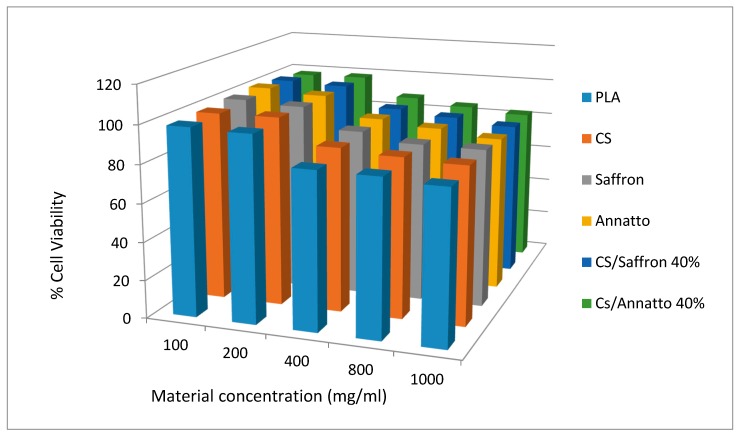
HUVEC cells viability after incubation for 24 h for different concentrations of CS, saffron, annatto, and their encapsulated nanopreparations, compared to biocompatible PLA polyester.

Even though chitosan is extensively used as a drug carrier due to its low cytotoxicity, saffron and annatto cytotoxicity were also tested in order to ensure that they are appropriate for the proposed applications. In Figure 6, the HUVEC cells’ viability is presented after incubation for 24 h of neat CS, saffron, annatto, and their nanoencapsulated additives, in comparison with a well-established biocompatible polymer (poly(lactic acid), PLA), which is extensively used in biomedical applications [52,53]. 

From the calculated data, it can be seen that neat materials (CS, saffron, and annatto) have in all studied concentrations similar cytotoxicity to PLA. This was expected since all of these materials are naturally occurring with many applications in food and pharmaceutical industries, especially CS. Similar behaviour was also observed for the encapsulated annatto and saffron additives into chitosan nanoparticles. In all concentrations, their cytotoxicity was comparable to that of neat materials. Thus, it can be said that these materials can be used in biomedical applications.

### 2.2. Sunscreen Emulsions

For the preparation of the sunscreen emulsions, the followed procedure resulted in the formation of cream-like emulsions that had a yellowish color in the case of annatto and UF annatto and a yellow to orange color in the case of saffron and UF saffron preparations. Additionally, increasing amounts of annatto and UF annatto resulted in an increase of color intensity, as expected, while blank (with no sunscreen protecting agent) emulsions, prepared also for comparison, had a white to creamy-white color.

#### 2.2.1. Emulsion Stability Results

In regards to emulsion stability, monitoring the pH value during storage is a crucial aspect, since pH changes indicate the occurrence of possible chemical reactions [54,55]. Given that the human skin pH value ranges from 4.5 to 6.0, products intended for topical use should have a pH value that is within that range [56]. In the present study, all prepared formulations had a pH value well within this range (pH value ranged from 5.44 to 5.88), indicating that they can be used as topical sunscreen emulsions. 

**Figure 7:**
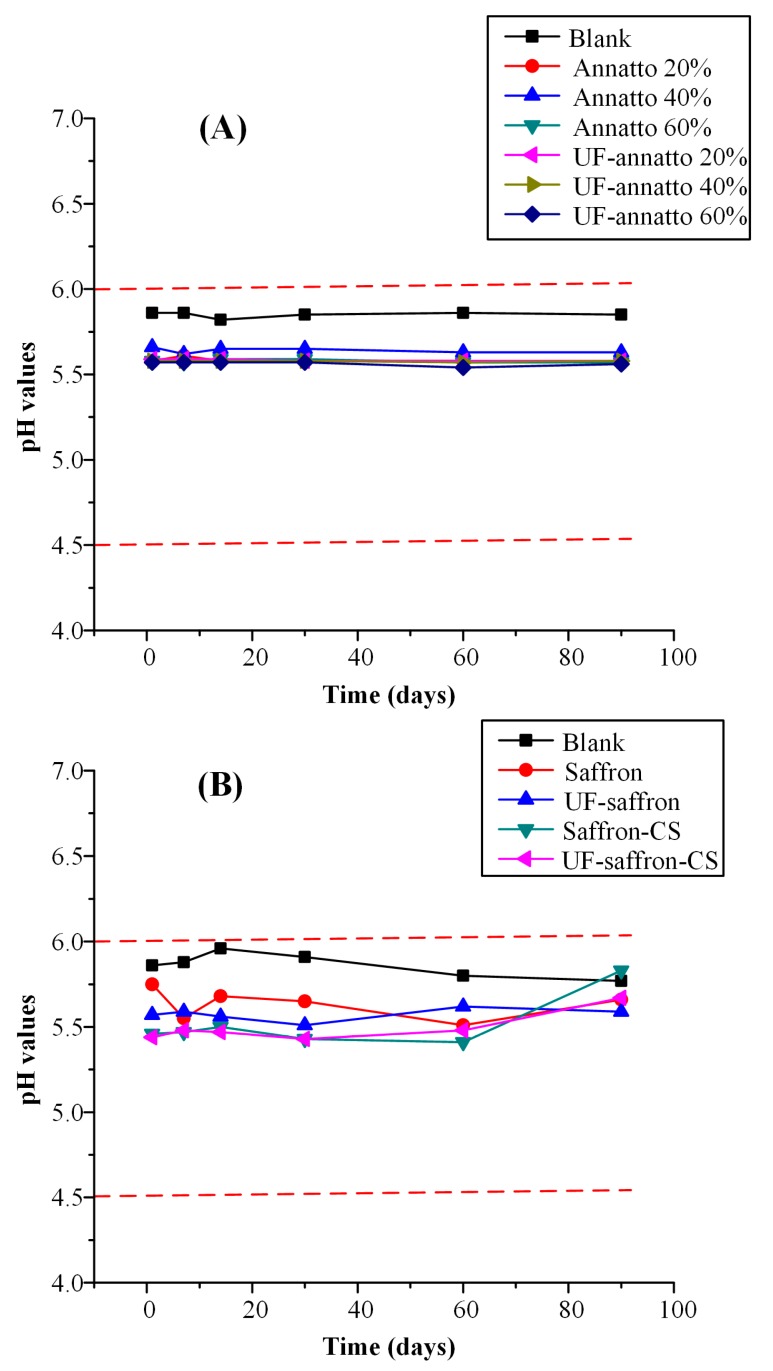
pH values of prepared sunscreen emulsions during storage stability studies for annatto/UF-annatto (**A**) and saffron/UF-saffron (**B**) samples. Red dashed lines depict the acceptable pH range of 4.5–6.0.

Figure 7 shows the pH values during storage stability for all formulations. In regards to annatto- and UF-annatto-based formulations, all emulsions had a slightly lower pH value compared to the blank emulsion, which, however, was within the acceptable pH range, and good pH stability was observed up to 90 days in all cases. Additionally, good pH emulsion stability was also observed for the saffron and UF saffron emulsions independently of the use of CS and the preparation of nanoparticles, indicating that the incorporation of the sunscreen agents within a CS-based nanoparticle system does not affect the pH stability of the resultant emulsion.

**Figure 8:**
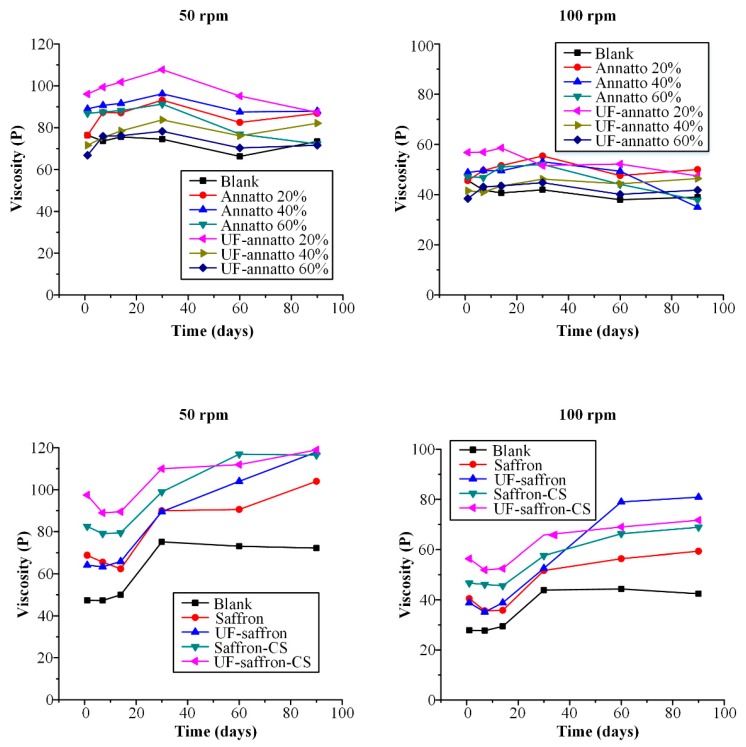
Viscosity profile of prepared sunscreen emulsions during storage stability studies.

Another important factor related to emulsion stability is the changes that may occur in emulsion’s viscosity profile. Changes in viscosity during storage may result in several defects, not only in regards to aesthetic appearance (liquefaction) but also in the fundamental aspects of the final product, such as the sun-protective ability of the emulsion. Therefore, Figure 8 shows the viscosity stability profile during storage of all prepared formulations for up to 90 days.

In the case of annatto-based sunscreen emulsions, the CS nanoparticles containing 20% wt. of UF annatto showed the highest viscosity in either 50 or 100 rpms. In general, most of the prepared emulsions showed increased viscosity during stability, which may be attributed to the presence of both annatto (or UF annatto) and CS. This is a desirable characteristic of sunscreen formulations since higher viscosity values can result in better photoprotection efficacy, as the consumer usually tends to apply a thicker layer of the product, culminating in a more effective film (higher SPF) [57]. Analytically, in the case of 50 rpms, all annatto and UF-annatto–CS nanoparticle emulsions showed an increase (less than 10%) in viscosity up to 30 days, and then a small decrease was observed up to 90 days (again less than 10% from the initial value), indicating that the prepared formulations show good stability in terms of stability. Similar results were observed in the case of 100 rpm, except in the case of 40% and 60% wt. annatto–CS nanoparticle emulsion, where a higher than 10% decrease in viscosity was observed after 90 days of storage. This is a common behavior of sunscreens containing a higher percentage of sunscreen agent added in the form of powder, which leads in flocculation of the emulsion [58].

In the case of saffron- and UF-saffron-based sunscreen emulsions, results showed a clear increase in viscosity compared to the blank emulsions in both 50 and 100 rpm. Additionally, the use of CS-loaded nanoparticles resulted in higher viscosities compared to emulsions containing neat saffron or UF saffron, indicating that the prepared nanoparticles led to a further increase in the emulsion’s viscosity. As in the case of annatto-based emulsions, the UF-processed saffron showed the highest initial viscosity in both 50 and 100 rpm. All saffron- and UF-saffron-prepared emulsions showed a small decrease in viscosity for up to 14 days followed by a sharp increase in 30 days, while in the case of 50 rpm, neat saffron and UF saffron emulsion viscosities increase constantly up to 90 days, indicating that the incorporation of the sunscreen agents within the CS nanoparticle system results in improved rheological stability. Similar results were observed in 100 rpm, where the neat UF saffron emulsion showed a ~100% increase in viscosity after 90 days of storage, compared to the UF-saffron–CS nanoparticle emulsion, where the increase in viscosity was less than 10%. 

The stability of the prepared emulsions was also assessed using the freeze at −4 °C and defreeze at 25 °C test, which is a common test in order to simulate the long-term shelf-life stability. Results showed that no phase separation occurred during these stability tests, while no significant viscosity changes were recorded (data not shown). Hence, it can be concluded that the prepared emulsions have excellent long-term shelf-life stability, while their characteristics are unaffected by temperature variations.

Additionally, color stability of the prepared emulsions was assessed over a period of 90 days using reflectance colorimetry. Results in Table 2 show no signs of color change, confirmed by the very close lightness (L), redness (a*), and yellowness (b*) values of the emulsions.

#### 2.2.2. Emulsion SPF

The efficacy of the prepared sunscreen emulsions in regards to UV radiation protection properties was expressed by the SPF value. Generally, sunscreen products with SPF values of 2–12 provide minimum sunscreen protection, SPF of 12–30 provides moderate protection, while products with SPF > 30 provide high protection. Table 3 shows the calculated SPF values for the prepared sunscreens along with the blank emulsion. SPF values of all products containing a sunscreen agent varied from 2.15 to 4.85, indicating that in all cases, a minimum sunscreen protection is achieved compared to blank emulsion (SPF = 1.00). In regards to annatto- or UF-annatto-based emulsions, increasing sunscreen agent concentration did not result in any increase against UV radiation protection (expressed in terms of SPF values), while in the case of saffron and UF saffron, the use of UF saffron resulted in doubled SPF values, indicating that the purification process of saffron resulted in emulsions with improved sunscreen protection.

The increased sunscreen protection conferred by annatto and saffron can be attributed to the presence of a number of ethylene double bonds in their structures which can absorb UV radiation in the same way as a typical synthetic fluorescent brightening agent, e.g., stilbene derivative, does.

## 3. Materials and Methods

### 3.1. Materials

For the preparation of nanoparticles, CS with high molecular weight (M_W_: 350,000 g/moL, deacetylation degree >75%, and viscosity 800–2000 cps), poly(L-lactide) (PLA) with viscosity ~1.0 dL/g (Mn = 59,000 g/mol), and sodium triphosphate (TPP) were supplied by Aldrich chemicals. Annatto and UF annatto in powder form were received from Alps Industries Ltd. (Tronica City, Ghaziabad, Uttar Pradesh 201102, India), while saffron was received from Crocos Cooperation of Kozani (Kozani, Greece). For the preparation of sunscreen emulsion, olive oil, sesame oil, ethylhexylglycerin, shea butter, glycerin, cetostearyl alcohol, cetyl alcohol, sodium citrate, beeswax, xanthan gum, polysorbate 60, steatic acid, triglycerides, and phenoxyethanol were kindly donated from Novita Group (Thessaloniki, Greece). All other materials and reagents used in this study were of analytical grade of purity.

### 3.2. Extraction and Ultrafiltration of Saffron

For the extraction of saffron, Crocos was placed into a beaker with deionized water (2% *w*/*v*) and warmed up for almost 3 h at 80–90 °C with simultaneous stirring. The resultant suspension was kept until next morning at room temperature in the dark in order to have a complete extraction. The aqueous phase was selected and dried until the dry ground saffron was obtained. For the UF process, a laboratory ultrafiltration unit equipped with a tubular membrane supplied by PCI Membranes (Fareham, Hampshire, UK) was used throughout our work. The membrane used for the UF process was the ES404, a polyethersulphone type membrane supplied by PCI Membranes (UK). The ultrafiltration process for 1.8 L of a liquid extract (2%) of saffron was carried out at ~35 °C and 10 bar pressure. At the stage of diafiltration, 1.5 L of water (in portions of 250 mL) was added and the process was continued until the concentration of the initial volume was at ~750 mL. The permeate was dried and purified UF saffron was received.

### 3.3. Preparation of CS Nanoparticles

CS nanoparticles were prepared according to the ionotropic gelation method [59]. Blank nanoparticles were obtained upon the addition of TPP aqueous solution to a CS acetic acid solution at a CS-to-TPP ratio of 2:1. The formation of nanoparticles was a result, as previously reported [60], from the interaction between the negative groups of TPP and the positively charged amino groups of CS. For the preparation of annatto or saffron-loaded nanoparticles, an aqueous solution of the substances was added to the prepared CS solution. Three different annatto and UF annatto (i.e., 20%, 40%, and 60% *w*/*w*) and one for saffron and UF saffron (i.e., 33% *w*/*w*) concentrations were tested based on CS quantity. Ultracentrifugation was applied at 13,000 rpm for 10 min in order to collect the nanoparticles, which were then purified twice with deionized water. The purified nanoparticles were then frozen and lyophilized using a freeze dryer system (Scanvac Coolsafe, Labogen Scandinavia, Blegistrasse, Baar, Switzerland) for 4 days at about −100 °C under vacuum in order to obtain the final dried nanoparticle product.

### 3.4. Characterization of CS Nanoparticles

#### 3.4.1. Morphological Characterization of Nanoparticles

The morphology of the prepared nanoparticles was studied using scanning electron microscopy (SEM) (JEOL JSM 6390 and JSM 840A apparatus (Oxford Instruments, Tubney Woods Abingdon, Oxfordshire, UK). The samples were covered with a carbon coating in order to provide good conductivity for the electron beam. Operating conditions were: Accelerating voltage 20 kV, probe current 75 nA, and counting time 60 s.

#### 3.4.2. Size Measurements of Nanoparticles

The particle size distribution of prepared nanoparticles was determined by dynamic light scattering (DLS) using a Zetasizer Nano Instrument (Malvern Instruments, Nano ZS, ZEN3600, Malvern, UK) operating with a 532-nm laser. A suitable amount of nanoparticles was dispersed in distilled water, creating a total concentration 1% *v*/*v* and was kept at 37 °C under agitation at 100 rpm.

#### 3.4.3. Wide Angle X-ray Diffractometry (WAXD)

WAXD was used to investigate the physical form (crystalline or amorphous) of drug dispersion within the CS matrix of the nanoparticles. The WAXD experiments were performed from 5 to 60° using a MiniFlex II XRD system from Rigaku Co. (Chalgrove, Oxford, UK) with Cu Ka radiation (l = 0.154 nm).

#### 3.4.4. Fourier Transformation Infrared Spectroscopy (FTIR)

FTIR spectra were obtained using a Perkin-Elmer FTIR spectrometer (model Spectrum 1000, Dresden, Germany). In order to collect the spectra, a small amount of freeze-dried nanoparticles was mixed with KBr (1 wt% nanoparticles) and compressed to form tablets. The IR spectra of these tablets, in absorbance mode, were obtained in the spectral region of 450–4000 cm^−1^ using a resolution of 4 cm^−1^ and 20 coadded scans.

#### 3.4.5. Thermogravimetric Analysis (TGA)

Thermogravimetric analysis (TGA) of neat and encapsulated samples were carried out using an SETARAM SETSYS TG-DTA 16/18 instrument (Lyon, France) by heating the samples from 25 to 600 °C in a 50 mL/min flow of N_2_ at a heating rate of 20 °C/min.

#### 3.4.6. Evaluation of Additive Encapsulation

The non-entrapped annatto quantity (free annatto) was measured in the clear supernatant collected after nanoparticle centrifugation (20,000 rpm for 30 min at 25 °C) using UV spectrometry (Shimadzu PharmaSpec UV-1700, Tokyo, Japan) at 400 nm. The corresponding calibration curves were produced using the supernatant of blank nanoparticles. Nanoparticle yield, annatto loading, and entrapment efficiency (EE) were calculated from these equations, respectively:

Yield (%) = (nanoparticle weight) × 100/(weight of polymer and annatto initially)(1)

Loading (%) = (annatto weight in nanoparticles) × 100/(weight of nanoparticles)(2)

EE (%) = (annatto weight in nanoparticles) × 100/(weight of annatto)(3)

Nanoparticle weight was estimated after freeze drying of centrifuged nanoparticles.

#### 3.4.7. In Vitro Cytotoxicity Study

The cytotoxicity of neat and encapsulated materials, in comparison to PLA, was evaluated by measuring the viability of HUVE cells in the presence of different concentrations of studied materials. Cell viability was determined by the [3-(4,5-dimethylthiazol-2-yl)-2,5-diphenyl tetrazolium bromide] (MTT) assay. HUVEC were seeded in 24-well plates at a density of 30,000 cells per well in 500 μL cell culture medium. Twenty-four hours after plating, different amounts of studied materials (suspended in culture medium) were added in the wells. After 24 h of incubation at 37 °C, 50 μL of MTT solution (5 mg/mL in PBS pH 7.4) was added into each well and plates were incubated at 37 °C for 2 h. The medium was withdrawn and 200 μL acidified isopropanol (0.33 mL HCl in 100 mL isopropanol) was added in each well and agitated thoroughly to dissolve the formed crystals. The solution was transferred to 96-well plates and immediately read on a microplate reader (Biorad, Hercules, CA, USA) at a wavelength of 490 nm. The experiments were performed in triplicate. For each material, five different concentrations were tested, namely, 100, 200, 400, 800, and 1000 mg/mL. The biocompatibility of studied materials was expressed as % cell viability, which was calculated from the ratio between the number of cells treated with the tested formulations and that of nontreated cells (control).

### 3.5. Preparation of Emulsions

O/W emulsions were prepared containing 0.2% *w*/*v* of annatto (or UF annatto)- or saffron (or UF saffron)-loaded CS nanoparticles. Briefly, annatto-loaded CS nanoparticles were homogenized in the water phase, which consisted of glycerin (4.7% *w*/*w*), xanthan gum (1.3% *w*/*v*), and citric acid (0.7% *w*/*v*) heated at 80 °C and homogenized with the oil phase, which consisted of olive oil (47.8% *w*/*w*) in the case of annatto and sesame oil (47.8% *w*/*w*) in the case of saffron, along with cetyl alcohol (8.7% *w*/*w*), cetostearyl alcohol (8.7% *w/w*), polysorbate 60 (8.7% *w*/*w*), shea butter (8.7% *w*/*w*), steatic acid (8.7% *w*/*w*), and beeswax (8.7% *w*/*w*). The resultant emulsions were left under stirring for approximately 2 h and then phenoxyethanol and ethylhexylglycerin were added.

### 3.6. Characterization of Emulsions

#### 3.6.1. Emulsion Stability

The stability of the resultant emulsion was monitored in terms of pH and viscosity after 7, 14, 30, 60, and 90 days of storage after preparation. pH measurements were conducted by dipping the pH sensor of a Microprocessor WTW pH 535 into the emulsion, while viscosity was measured at 50 and 100 rpm using the R3 spindle of a Visco Star Plus viscometer.

Additionally, freeze–thaw cycle testing study was conducted in order to evaluate the thermal stability of the prepared emulsions. The emulsions were put in the fridge at −4 °C for 24 h and then were removed from the fridge and conditioned at room temperature (25 °C) for 24 h. The above cycle was repeated five times. The emulsions were assessed visually in order to evaluate if any separation effects occurred, while viscosity measurements were also conducted.

Finally, the color stability of the prepared emulsions was assessed over a period of 90 days using reflectance colorimetry. Measurements were performed using a Macbeth CE 3000 spectrophotometer (Macbeth, London, UK) under D65 illumination, 10 degrees standard observer with UV included and specular component excluded. The lightness (L), redness (a*), and yellowness (b*) values of the emulsions were measured in order to assess the color stability of the emulsions.

#### 3.6.2. SPF Determination

SPF was determined using the diluted solution transmittance method. Briefly, all samples were weighed (1 g), transferred to a 100-mL volumetric flask, diluted to volume with ethanol, mixed for 5 min, and then filtered through Whatman filters. A 5-mL sample was transferred to a 25-mL volumetric flask and diluted to volume with ethanol. The absorption values were obtained in the range of 290–320 nm (every 5 nm) and three determinations were made at each point. Then, the Mansur equation was used to determine the SPF values of the formulations:

(4) $$\mathrm{SPF}=\mathrm{CF}\times \sum_{320}^{290}\mathrm{EE}\left(\lambda \right)\times I\left(\lambda \right)\times \mathrm{abs}\left(\lambda \right)$$

where CF = 10 (correction factor), EE (λ) = erythemogenic effect of radiation at wavelength λ, I(λ) = intensity of solar light at wavelength λ, and abs(λ) = absorbance of sample at wavelength λ. The values for the term “EE × I” are constants, which were determined by Sayre et al. [61].

## 4. Conclusions

CS-based nanoparticles were prepared with all tested sunscreen agents (annatto, UF annatto, saffron, and UF saffron). SEM analysis revealed that formed nanoparticles had a spherical and irregular shape, while their size varied from ~150 to ~500 nm. Additionally, XRD analysis showed amorphous dispersion in the case of annatto and UF annatto and crystalline dispersion in the case of saffron and UF saffron nanoparticles, while FTIR analysis showed the formation of H-bond interactions. Sunscreen emulsions prepared from the resultant CS–sunscreen agent(s) nanoparticles showed good storage stability for up to 90 days at room temperature in terms of pH and viscosity, while minimum sunscreen protection was determined with SPF values varying from 2.15 to 4.85 in all cases. All materials have high color stability as well as low cytotoxicity.

## Figures and Tables

**Table 1:** Nanoparticles particle size (D(50)), zeta-potential, yield, sunscreen agent loading, and entrapment efficiency results.

Sample	D(50) (μm)	Zeta Potential (mV)	Yield (%)	Loading (%)	Entrapment Efficiency (%)
**Blank**	250 ± 7	36 ± 3	-	-	-
**Annatto** **–** **CS**					
20 wt%	287 ± 5	34 ± 3	70.83	15.62	66.40
40 wt%	310 ± 8	41 ± 3	65.80	29.62	68.25
60 wt%	340 ± 11	46 ± 3	61.25	41.17	67.25
**UF-annatto** **–** **CS**					
20 wt%	263 ± 9	35 ± 3	47.80	15.69	45.01
40 wt%	284 ± 10	40 ± 3	46.34	38.27	62.06
60 wt%	303 ± 8	47 ± 3	55.07	48.71	71.54
**Saffron** **–** **CS**	321 ± 6	42 ± 3	67.75	20.52	21.25
**UF-saffron** **–** **CS**	298 ± 8	43 ± 3	83.75	25.61	37.50

**Table 2:** Chromatic co-ordinate values of lightness (L), redness (a*), and yellowness (b*) for the emulsions of saffron and annatto.

	L		a*		b*	
	Day 1	Day 90	Day 1	Day 90	Day 1	Day 90
**CS–Saffron**	89.58	89.17	−0.34	−0.37	2.77	2.68
**CS–UF-Saffron**	88.15	88.51	−0.39	−0.41	2.64	2.69
**CS–Annatto**	90.01	89.95	−0.40	−0.44	2.51	2.46
**CS–UF-Annatto**	91.11	91.18	−0.42	−0.45	2.49	2.51

**Table 3:** Sun protection factor (SPF) values of the prepared emulsions.

Sample	SPF Value
**Blank**	1.00
**Annatto** **–** **CS**	
20 wt%	2.63
40 wt%	2.27
60 wt%	2.24
**UF-annatto** **–** **CS**	
20 wt%	2.76
40 wt%	2.71
60 wt%	2.56
**Saffron** **–** **CS**	2.15
**UF-saffron** **–** **CS**	4.85
